# Activity-dependent dendrite patterning in the postnatal barrel cortex

**DOI:** 10.3389/fncir.2024.1409993

**Published:** 2024-05-17

**Authors:** Naoki Nakagawa, Takuji Iwasato

**Affiliations:** ^1^Laboratory of Mammalian Neural Circuits, National Institute of Genetics, Mishima, Japan; ^2^Graduate Institute for Advanced Studies, SOKENDAI, Mishima, Japan

**Keywords:** activity-dependent circuit formation, postnatal brain development, dendrite refinement, barrel cortex, Golgi apparatus, *in vivo* imaging, spontaneous activity

## Abstract

For neural circuit construction in the brain, coarse neuronal connections are assembled prenatally following genetic programs, being reorganized postnatally by activity-dependent mechanisms to implement area-specific computational functions. Activity-dependent dendrite patterning is a critical component of neural circuit reorganization, whereby individual neurons rearrange and optimize their presynaptic partners. In the rodent primary somatosensory cortex (barrel cortex), driven by thalamocortical inputs, layer 4 (L4) excitatory neurons extensively remodel their basal dendrites at neonatal stages to ensure specific responses of barrels to the corresponding individual whiskers. This feature of barrel cortex L4 neurons makes them an excellent model, significantly contributing to unveiling the activity-dependent nature of dendrite patterning and circuit reorganization. In this review, we summarize recent advances in our understanding of the activity-dependent mechanisms underlying dendrite patterning. Our focus lays on the mechanisms revealed by *in vivo* time-lapse imaging, and the role of activity-dependent Golgi apparatus polarity regulation in dendrite patterning. We also discuss the type of neuronal activity that could contribute to dendrite patterning and hence connectivity.

## Introduction

The sophisticated neural circuits underlying proper brain function in animals are first formed as coarse neuronal connections during embryonic development. Later, such immature connections are reorganized during postnatal stages, establishing a precise connectivity tailored to each brain area. Morphological and functional neuron remodeling, depending on neuronal activity evoked either spontaneously or by extrinsic stimuli, causes this postnatal circuit reorganization (Goodman and Shatz, 1993; Katz and Shatz, 1996; Wong and Ghosh, 2002). Particularly, activity-dependent remodeling of the dendritic pattern is key for circuit reorganization, whereby individual neurons rearrange and optimize their presynaptic partners. Dendrite refinement has been observed in various neuronal types in diverse brain regions and species, and is therefore considered a general mechanism for building functional neural circuits (Cline, 2001; Wong and Ghosh, 2002; Emoto, 2011).

The activity-dependent mechanisms underlying dendrite patterning have been studied in a wide range of models, including tectal neurons in Xenopus tadpole, retinal ganglion cells in chick and cat, olfactory bulb mitral cells in mouse, and cortical neurons in mouse, cat, and ferret (Harris and Woolsey, 1981; Katz and Constantine-Paton, 1988; Bodnarenko and Chalupa, 1993; Kossel et al., 1995; Wong et al., 2000; Matsui et al., 2013; Fujimoto et al., 2023). Among them, spiny stellate neurons, the major type of layer 4 (L4) excitatory neurons, in the primary somatosensory cortex (barrel cortex) of mice and rats have attracted attention as a model of activity-dependent dendrite patterning (Woolsey and Van der Loos, 1970; Erzurumlu and Gaspar, 2012; Iwasato and Erzurumlu, 2018).

**Figure 1:**
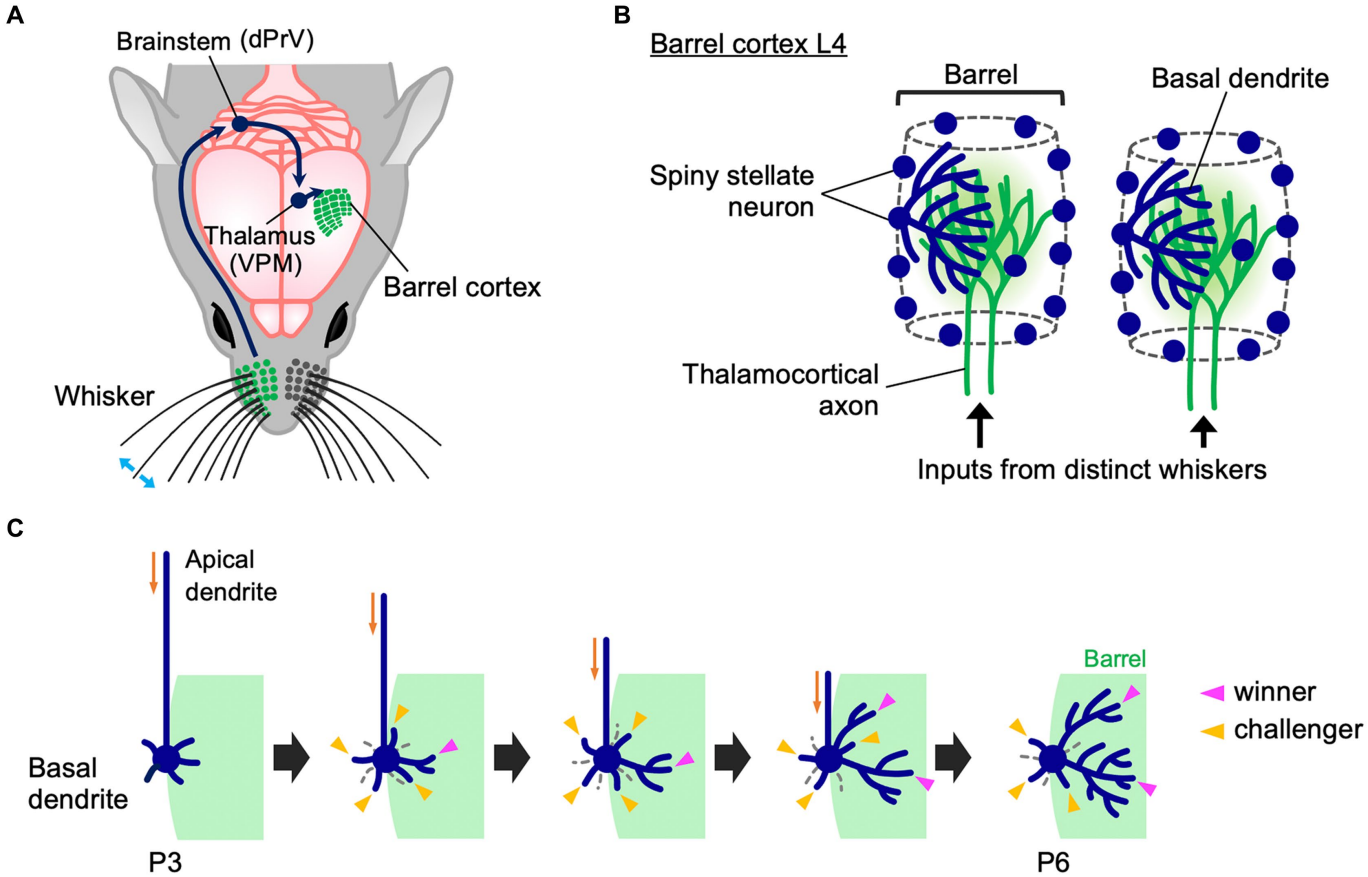
Whisker-barrel circuit and dendrite refinement of barrel cortex layer 4 spiny stellate neurons. **(A)** A schematic diagram of the mouse whisker-barrel system. The tactile information received by the whiskers is topographically conveyed to the contralateral barrel cortex layer 4 (L4) through the brainstem and the thalamus. dPrV: dorsal principal trigeminal nucleus, VPM: ventral posterior medial nucleus. **(B)** Barrel cytoarchitecture. In each barrel, the termini of thalamocortical axons (TCAs) that transmit sensory inputs from the corresponding single whiskers form distinct clusters in barrel cortex L4. Spiny stellate neurons, the major excitatory neurons in barrel cortex L4, are located primarily at the edge of TCA clusters, thereby showing the “barrel” shape. Spiny stellate neurons expand their basal dendrites selectively toward a corresponding single barrel to establish synapses with the corresponding TCA termini. Dendrites drawn only in some neurons for simplicity. **(C)** Dynamics of formation of asymmetric dendrite patterns in L4 spiny stellate neurons. At P3, a spiny stellate neuron has more dendritic trees in the inner domain (green) than in the outer domain, but inner and outer dendritic trees are equally primitive in morphology. Between P3 and P6, many short dendritic trees emerge and disappear both inside and outside the barrel (“challenger” dendritic trees, indicated by yellow arrowheads). During this extensive turnover, only a few trees (indicated by magenta arrowheads) are stabilized and elaborated to be “winners.” Importantly, winners are selected only from the challengers that emerge in the barrel-side (green). Note that late-born dendritic trees can become winners.

In rodents, facial whiskers are an important sensory organ whereby animals perceive their environment. Tactile stimuli to whiskers reach the barrel cortex L4 via the dorsal principal trigeminal (dPrV) nucleus in the brainstem and the ventral posterior medial (VPM) nucleus in the thalamus (Figure 1A) (Fox, 2008; Iwasato and Erzurumlu, 2018). In barrel cortex L4, the information from each whisker is processed by an array of neurons called “barrel,” whose arrangement represents the spatial pattern of whiskers in the face. Termini of thalamocortical axons (TCAs) that transmit inputs from a whisker are distributed only within the corresponding barrel. L4 spiny stellate neurons are preferentially located at the edge of each barrel and expand their basal dendrites asymmetrically toward the barrel center (Figure 1B) (Harris and Woolsey, 1981; Simons and Woolsey, 1984). This asymmetric dendritic projection pattern, formed in an activity-dependent manner essentially during the first postnatal week, underlies precise tactile information processing in rodents (Nakazawa et al., 2018; Iwasato, 2020; Nakagawa and Iwasato, 2023); therefore, understanding how this unique dendritic asymmetry is established during postnatal development is of importance.

In this review, we summarize recent advances in our understanding of the activity-dependent mechanisms underlying dendrite patterning and postnatal circuit reorganization based on studies using whisker-barrel circuits.

### Dendritic patterning of L4 spiny stellate neurons in the neonatal barrel cortex

The activity transmitted through TCAs is critical for dendritic patterning of barrel cortex L4 spiny stellate neurons. Removing glutamatergic synaptic transmission from TCA termini by knocking out both VgluT1 and VgluT2, two major vesicular glutamate transporters in the brain, in the sensory thalamus impairs formation of cortical layers, barrel maps, and L4 neuron dendrite morphology (Li et al., 2013). Similarly, when thalamocortical synaptic transmission is reduced by a thalamus-specific double knockout of RIM1 and RIM2, which regulate synaptic vesicle fusion, barrel formation and L4 neuron dendritic asymmetry become impaired (Narboux-Neme et al., 2012). These results suggest a critical role of TCA-derived activity for barrel circuit formation, including dendritic patterning of L4 neurons.

Gene knockouts also indicated that the dendrite refinement of L4 spiny stellate neurons relies on the postsynaptic N-methyl-D-aspartate-type glutamate receptor (NMDAR) activity induced by presynaptic thalamocortical inputs. Knockout of either NR1 or NR2B subunits of NMDAR causes impaired asymmetry of dendritic projections (Iwasato et al., 2000; Datwani et al., 2002; Espinosa et al., 2009; Mizuno et al., 2014). NMDAR is a tetrameric complex composed of two NR1 subunits and two NR2 subunits; NR1 is the essential subunit and NR2B the modulatory subunit, dominant in the neonatal brain (Nakanishi, 1992; Mori and Mishina, 1995). Genetic approaches in mice identified dozens of molecules related to synaptic transmission and its downstream signaling cascade implicated in barrel formation and dendrite refinement of L4 spiny stellate neurons. Such genes include metabotropic glutamate receptor 5, protein kinase A (PKA), PKA-anchoring protein 5, adenylyl cyclase 1, phospholipase C-β1, Ras GTPase-activating proteins, Fibroblast growth factor receptors, Tropomyosin receptor kinase A, BTB/POZ domain-containing 3, LIM domain-only 4, neurogenic differentiation 2, retinoic acid-related orphan receptor alpha (RORα) and RORβ (Abdel-Majid et al., 1998; Hannan et al., 2001; Barnett et al., 2006; Inan et al., 2006; Ince-Dunn et al., 2006; Kashani et al., 2006; Watson et al., 2006; Iwasato et al., 2008; Lush et al., 2008; She et al., 2009; Jabaudon et al., 2012; Matsui et al., 2013; Ballester-Rosado et al., 2016; Huang et al., 2017; Huang and Lu, 2018; Vitalis et al., 2018; Zhang et al., 2019; Clark et al., 2020; Rao et al., 2022). Using mitral cells in the mouse olfactory bulb, Imai and colleagues recently reported that strong NMDAR activation in prospective winner dendrites locally suppresses RhoA activity, protecting the dendrite from depolarization-induced, neuron-wide RhoA activation, which acts as a dendrite retraction signal (Fujimoto et al., 2023). This system also works in barrel cortex L4 neurons (Fujimoto et al., 2023).

### Mechanisms of dendritic patterning revealed by *in vivo* time-lapse imaging

It is generally assumed that spiny stellate neurons in barrel cortex L4 exhibit symmetrical dendritic patterns during early neonatal stages but subsequently acquire asymmetrical dendritic patterns by simply eliminating outer dendrites and adding new inner dendrites and/or elaborating existing inner dendrites (Greenough and Chang, 1988; Espinosa et al., 2009; Emoto, 2011; Iwasato, 2020). This view was challenged by *in vivo* imaging approaches in the neonatal mouse cortex (Mizuno et al., 2014; Nakazawa et al., 2018; Iwasato, 2020; Wang et al., 2023). In these studies, L4 neurons were sparsely labeled and each was imaged repeatedly in the neonatal barrel cortex using two-photon microscopy.

In the mature barrel cortex, L4 excitatory neurons are classified by the absence and presence of apical dendrites into spiny stellate and star pyramid neurons, respectively (Simons and Woolsey, 1984; Lübke et al., 2000; Staiger et al., 2004). Importantly, spiny stellate neurons, the major L4 neurons, show asymmetric dendritic patterns but star pyramid neurons have symmetric dendrites. However, at early postnatal stages such as postnatal day 3 (P3), prospective spiny stellate neurons also have an apical dendrite, which hampers distinguishing spiny stellate neurons from star pyramid neurons by conventional histological analyses in brain slices. On the other hand, longitudinal *in vivo* imaging of the same neurons in the brain allows retrospective identification of prospective spiny stellate neurons in early postnatal development by their morphological features at later developmental stages such as P6; thus allowing to analyze the dendritic morphology of spiny stellate neurons in early postnatal stages (Nakazawa et al., 2018; Iwasato, 2020).

Longitudinal *in vivo* imaging of L4 neurons in the mouse barrel cortex revealed that at P3, a spiny stellate neuron has a larger number of inner dendritic trees, which originate from the barrel-side half of the soma, than outer dendritic trees (Nakazawa et al., 2018). However, at this age both inner and outer dendritic trees are equally primitive in morphology (Figure 1C). Between P3 and P6, the ratio of inner to outer dendritic trees does not change. However, during this period, dendritic trees show extensive turnover both inside and outside the barrel. Many newly emerged dendritic trees (i.e., “challenger” dendritic trees) quickly disappear. Among them, only a few are stabilized and elaborated and become winners. Importantly, these winners are primarily selected from challengers emerging inside the barrel. L4 spiny stellate neurons have multiple winner dendritic trees, and even late-born dendritic trees can become winners (Nakazawa et al., 2018). Thus, L4 spiny stellate neurons establish highly asymmetric dendritic patterns not by eliminating outer dendritic trees and adding new inner trees and/or elaborating existing inner trees during neonatal stages. In contrast, L4 spiny stellate neurons produce many challenger dendritic trees in various directions, and only a few winners are selected from the challengers that emerge in the appropriate direction. These winners are then stabilized and elaborated (Figure 1C).

Most L4 spiny stellate neurons, which are located at the barrel edge, can receive appropriate TCA inputs only from a specific direction toward the barrel center. On the other hand, L4 spiny stellate neurons located in the barrel center can receive appropriate TCA inputs from any direction. Such barrel-center spiny stellate neurons show much lower dendritic tree turnover than barrel-edge spiny stellate neurons (Nakazawa et al., 2018). In barrel-center spiny stellate neurons, most dendrites are stable and mildly grow, establishing dendritic projections without orientation bias. Thus, spatially biased presynaptic TCA inputs may play a key role regulating dendritic dynamics and dendritic orientation. When appropriate presynaptic partners are available only in a specific direction, neurons produce many dendritic trees in all directions, and select a few winners among dendritic trees that emerge in the right direction.

A more recent *in vivo* imaging study, using 1-h interval for imaging of barrel cortex L4 neuron dendrites in the neonatal mouse (Wang et al., 2023), further supports this view. This high time-resolution imaging allows to accurately identify the same dendritic branches across imaging sessions, which is often difficult with the imaging intervals (8 h) used in previous experiments (Mizuno et al., 2014; Nakazawa et al., 2018). This new study found that many dendritic branches (and trees) emerge and are eliminated even within a few hours. Both inner and outer dendritic branches (and trees) emerge and are eliminated with no clear difference in frequency. These results suggest that despite dendritic trees and branches being highly dynamic during neonatal stages, most of these rapid changes in dendritic morphology do not directly contribute to the formation of asymmetric dendritic patterns in spiny stellate neurons. Thus, L4 spiny stellate neurons establish highly asymmetric dendritic patterns through extensive trial-and-error emergence/elongation and elimination/retraction of dendritic trees and branches rather than simple emergence/elongation of inner dendritic trees/branches and elimination/retraction of outer dendritic trees/branches (Figure 1C).

### Golgi polarity in thalamocortical activity-dependent dendritic patterning

As described above, during dendrite refinement of barrel cortex L4 spiny stellate neurons, only a fraction of dendritic trees that emerge inside the barrel is selected as winners from a large number of transient dendritic trees (challenger dendritic trees) generated during continuous turnover both inside and outside the barrel (Figure 1C). Why do inner dendritic trees, but not outer dendritic trees, become winners?

**Figure 2:**
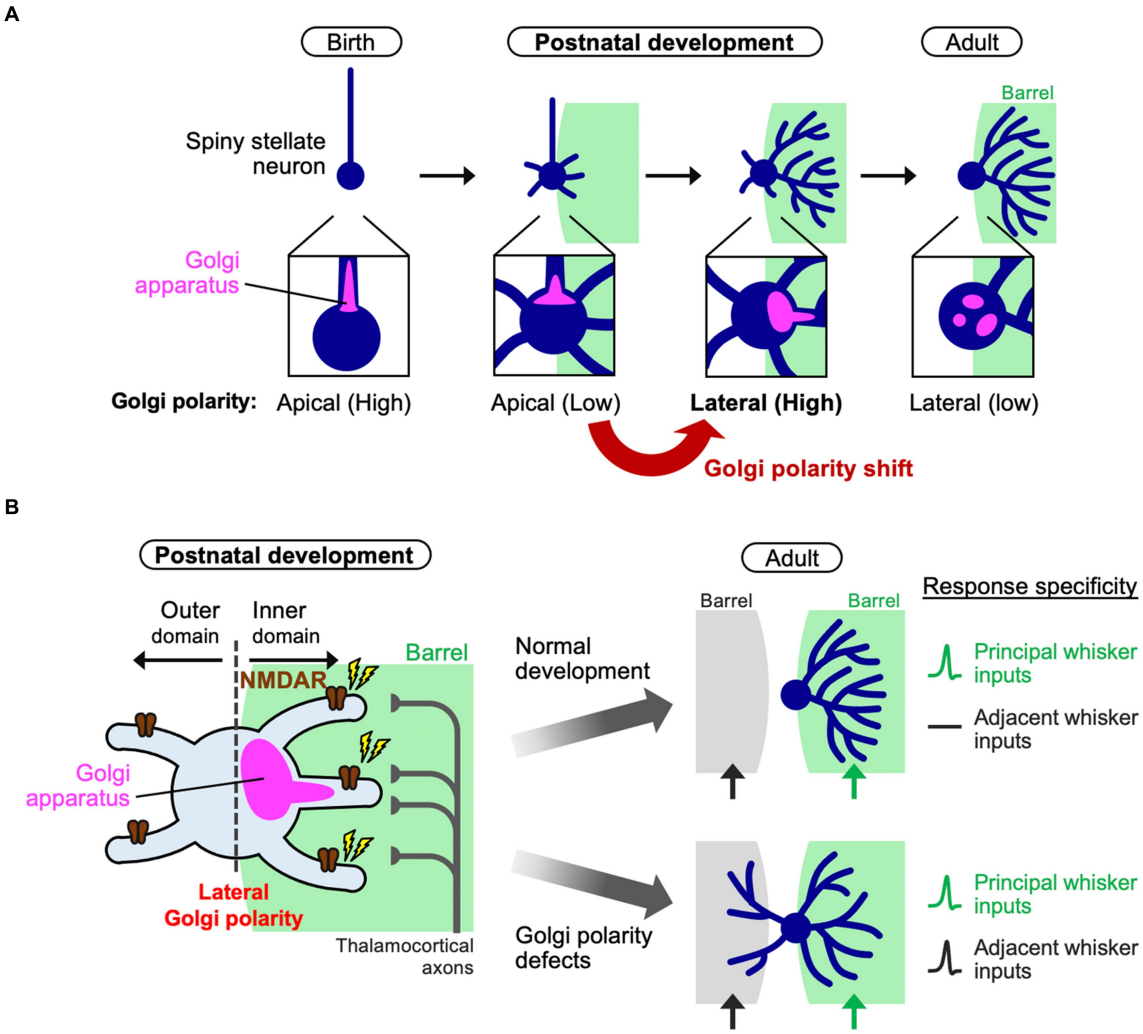
Polarity shift of the Golgi apparatus instructs dendrite refinement. **(A)** In barrel cortex L4, spiny stellate neurons initially have apical Golgi polarity. During postnatal development (the first postnatal week), spiny stellate neurons de-construct the initial polarity and shift it to the lateral direction oriented toward a single barrel. Finally, after completing of circuit reorganization, spiny stellate neurons decrease the lateral Golgi polarity (adult). **(B)** During dendrite refinement in a spiny stellate neuron, NMDA receptors (NMDARs) are activated by thalamocortical inputs from a single barrel. The Golgi apparatus translocates toward the subcellular domain where NMDARs are activated [see also **(A)**]. Then, a few inner dendritic trees harbor the Golgi (at their base or inside) to be winners. In this way, the spiny stellate neuron establishes the asymmetric dendritic patterns, underlying the specific response to a single principal whisker (right, normal development). On the other hand, if the lateral Golgi polarization is impaired, the neuron has lower dendrite asymmetry, so that it responds to both principal and adjacent whiskers, compromising the whisker-dependent tactile discrimination in mice.

Recent evidence indicated a significant contribution of cell polarity in inner dendritic tree-specific winner emergence (Nakagawa and Iwasato, 2023). In fact, the Golgi apparatus distribution changes in L4 spiny stellate neurons during the neonatal stage. The Golgi apparatus in L4 spiny stellate neurons is positioned in the apical domain in early neonatal stages such as P3, but it is subsequently translocated and polarized to the lateral domain and oriented toward a single barrel by P5 (Figure 2A). This “lateral Golgi polarity” temporally matches with the progression of dendrite refinement: lateral polarity peaks on the active refinement stage (P5–P7) and disappears upon refinement completion (~P15). In contrast to spiny stellate neurons, star pyramid neurons do not show lateral Golgi polarity. The lateral Golgi polarity in L4 spiny stellate neurons relies on NMDAR activation, serving as an intracellular machinery that connects thalamocortical activity to dendrite patterning. Perturbing Golgi polarity results in less asymmetric dendrite patterning and lower response specificity to principal whisker stimulation.

How can a biased Golgi distribution in a neuron explain the fate of individual dendritic trees? The Golgi apparatus is a hub for intracellular vesicle transport and contributes to dendrite extension and elaboration (Horton et al., 2005; Ye et al., 2007). Indeed, during dendrite refinement in L4 spiny stellate neurons, the dendritic trees that harbor the Golgi at their base or inside are more elaborated than those without Golgi allocation (Nakagawa and Iwasato, 2023). Therefore, the laterally polarized Golgi distribution in neurons, biased toward the single barrel, may provide the chance of Golgi allocation only to inner dendritic trees and make them winners (Figure 2). It is likely that due to the physical capacity of the Golgi apparatus, when a few dendritic trees become winners, other dendritic trees can hardly be supported by the Golgi, making these trees short and/or transient, even in case of inner trees. As presynaptic TCAs are clustered in the barrel center, L4 neurons should make synapses predominantly on inner dendrites (Figure 2B), creating NMDAR signaling gradients and generating Golgi lateral polarity. On the other hand, losing NMDAR activity from a cell impairs lateral polarity of the Golgi apparatus (Nakagawa and Iwasato, 2023). In this situation, no dendrites become winners and most, both inside and outside the barrel, mildly grow, impairing the one-to-one functional relationship between a whisker and a barrel (Figure 2B).

### Thalamocortical activity in the neonatal barrel cortex

Dendritic refinement of L4 spiny stellate neurons largely relies on thalamocortical inputs (Narboux-Neme et al., 2012; Li et al., 2013). Although, in neonatal stages such as P6, barrel cortex L4 is innervated not only by TCAs but also by subplate neuron neurites, these subplate neurons also receive excitatory thalamocortical inputs (Higashi et al., 2002; Piñon et al., 2009; Kanold, 2019). In other words, during early postnatal period, L4 neurons are activated by thalamic inputs directly or indirectly. Therefore, it is important to know the type of activity that TCAs transmit to barrel cortex during neonatal stages and where in the trigeminal pathway this activity arises from.

Spontaneous correlated activity plays critical roles in the refinement of neuronal circuits in the sensory systems of developing mammals (Katz and Shatz, 1996; Kirkby et al., 2013; Martini et al., 2021; Nakazawa and Iwasato, 2021). In barrel cortex L4 of the neonatal mouse, there is spontaneous activity with a unique spatiotemporal pattern (Mizuno et al., 2018). L4 neurons that belong to the same barrel fire together, while those in different barrels fire in a different timing, providing the barrel-corresponding “patchwork” pattern to spontaneous activity. This patchwork-type spatiotemporal pattern of spontaneous activity is observed in the barrel cortex L4 during early postnatal stages such as P0 and P5 but not later (Mizuno et al., 2018; Nakazawa et al., 2020). L4 neurons around P9 show broadly synchronized activity across barrel borders, and by P11, cortical spontaneous activity is desynchronized (Nakazawa et al., 2020; Nakazawa and Iwasato, 2021).

Patchwork-type spontaneous activity is also observed in TCA termini during the first postnatal week (Mizuno et al., 2018); in fact, chemogenic silencing of the thalamus hampers detection of spontaneous activity in the cortex (Nakazawa et al., 2020). These findings suggest that patchwork activity is transmitted to cortical L4 neurons via TCAs. Cortical patchwork activity is also blocked by local anesthesia in the whisker pads but not by severing the infraorbital nerves (IONs) (Mizuno et al., 2018; Nakazawa et al., 2020). IONs are peripherally projecting processes of trigeminal ganglion (TG) neurons, which innervate the whisker follicles. These results suggest that the cortical spontaneous activity is generated in the periphery but downstream of IONs. Thus, it is highly likely that the spontaneous activity originates in the TG.

A recent study has established a calcium imaging system of the TG *ex vivo* and found that neurons in the whisker-innervated region of the TG fire spontaneously during neonatal stages (Banerjee et al., 2022). This activity is blocked when chelating extracellular calcium. Most firing neurons have medium-to-large diameter, and likely are mechanosensory neurons. Although TG neurons fire sparsely and have no clear spatiotemporal pattern, some neuron pairs with highly correlated firing tend to be located closely (Banerjee et al., 2022). Neurons that innervate the same whiskers are not clustered in the TG but close to each other (Erzurumlu and Jhaveri, 1992; da Silva et al., 2011; Banerjee et al., 2022). Therefore, it is possible that TG neurons that innervate the same whisker pad together tend to fire together. If so, this may generate the patchwork pattern corresponding to the barrel map in the barrel cortex. This hypothesis needs to be explored in future research.

Patchwork-type cortical activity is also generated by sensory feedback from self-generated whisker movements. Whisker and paw twitching are frequently observed in neonatal rodents during rapid eye movement (REM) sleep (Khazipov et al., 2004; Tiriac et al., 2012, 2014; Dooley et al., 2020). The sensory feedback from the twitching of the whiskers and paws appears to be a source of firing in the downstream trigeminal pathway. There is some degree of coupling between the twitching and the firing in the thalamus and cortex (Khazipov et al., 2004; Tiriac et al., 2012, 2014; Mizuno et al., 2018; Dooley et al., 2020). By using unit recording of the rat barrel cortex at P5, Blumberg and colleagues reported that about 12 and 23% of spontaneous whisker movements are accompanied with spindle bursts of barrels, during wake and REM sleep, respectively. They also reported that more than half of barrel activity is preceded by whisker twitches (Dooley et al., 2020). In this study, time-resolution was quite high and wake and sleep were precisely distinguished. By calcium imaging focusing on L4 neurons of the P5 mouse barrel cortex, Mizuno et al., demonstrated that about 11% of spontaneous whisker movements were associated with firing of L4 neurons within the corresponding barrel, and about 11% of L4 neuron firing episodes accompany spontaneous movements of the corresponding whisker (Mizuno et al., 2018). In this study, L4 neurons were identified accurately by using *in utero* electroporation-based cell labeling and TCA-red fluorescent protein (RFP) transgenic (Tg)-mediated L4 labeling. In addition, each barrel was precisely identified in a cellular level by TCA-RFP Tg-mediated barrel map labeling.

Sensory input also generates cortical activity. Rodents do not see or hear during neonatal stages because the retina and cochlea are not functional yet. On the other hand, in rodents, tactile sensation is already present, albeit partially, at birth. Although rodents do not show exploratory and whisking behaviors until around P12–P14 (for the mouse) (Arakawa and Erzurumlu, 2015; van der Bourg et al., 2017), even at birth, rodents already exhibit passive sensation from tactile organs, including the whiskers. Sensory inputs induced by whisker deflection are transmitted to the cortex via the brainstem and thalamus (Khazipov et al., 2004; Akhmetshina et al., 2016). The specific role of these three types of activity in neonatal animals in the refinement of barrel cortex circuits needs to be clarified in the future.

## Discussion

Recent studies using the mouse barrel cortex L4, have increased our understanding of the activity-dependent mechanisms of dendrite refinement and circuit reorganization. Mouse genetics studies have discovered dozens of molecules involved in the dendrite refinement of the barrel cortex L4 spiny stellate neurons. However, our knowledge on how these molecules are spatiotemporally coordinated within a neuron to determine the fate of individual dendritic trees is still lacking. This could be overcome by labeling endogenous molecules and their activities with subcellular resolution *in situ* and analyzing their spatiotemporal changes and correlation with the behavior of individual dendrites during refinement.

Apart from the function of individual molecules, an important viewpoint has been introduced, which is the dynamics of subcellular structures such as the Golgi apparatus (Nakagawa and Iwasato, 2023). Triggered by thalamocortical input, molecular activities should be converted to structural and functional changes of intracellular machinery, which drive morphological changes in neurons. Next, it will be necessary to elucidate the activity-dependent mechanisms underlying Golgi recruitment to specific dendrite(s) in L4 spiny stellate neurons, and to understand how the polarized Golgi enables asymmetric dendrite growth.

Continuous improvement of *in vivo* imaging approaches is important as well. Unlike conventional “snapshot” analysis by histology, *in vivo* imaging in living neonates allows us to directly understand the ongoing process of dendrite refinement. Moreover, dissecting the rules underlying the behavior of individual dendrites during refinement help clarify how the molecules and organelles work within a neuron.

Combining these multidisciplinary approaches is required for understanding the whole picture of the activity-dependent mechanisms underlying dendrite patterning, a critical step in postnatal neural circuit reorganization.

## Author contributions

NN: Writing – original draft, Writing – review & editing. TI: Writing – original draft, Writing – review & editing.
